# Knowledge domain and emerging trends in empagliflozin for heart failure: A bibliometric and visualized analysis

**DOI:** 10.3389/fcvm.2022.1039348

**Published:** 2022-11-03

**Authors:** Xuesong Zhang, Ying Zhang, Yuanhui Hu

**Affiliations:** ^1^Department of Cardiovascular Diseases, Guang’anmen Hospital, China Academy of Chinese Medical Sciences, Beijing, China; ^2^Department of Cardiovascular Diseases, Beijing Hepingli Hospital, Beijing, China

**Keywords:** empagliflozin, heart failure, CiteSpace, VOSviewer, bibliometrics, visualization

## Abstract

**Objective:**

Empagliflozin (EMPA), a sodium-glucose cotransporter 2 inhibitor (SGLT2i), is recommended for all patients with Heart failure (HF) to reduce the risk of Cardiovascular death, hospitalization, and HF exacerbation. Qualitative and quantitative evaluation was conducted by searching relevant literatures of EMPA for Heart Failure from 2013 to 2022, and visual analysis in this field was conducted.

**Methods:**

The data were from the Web of Science Core Collection database (WOSCC). The bibliometric tools, CiteSpace and VOSviewer, were used for econometric analysis to probe the evolvement of disciplines and research hotspots in the field of EMPA for Heart Failure.

**Results:**

A total of 1461 literatures with 43861 references about EMPA for Heart Failure in the decade were extracted from WOSCC, and the number of manuscripts were on a rise. In the terms of co-authorship, USA leads the field in research maturity and exerts a crucial role in the field of EMPA for Heart Failure. Multidisciplinary research is conducive to future development. With regards to literatures, we obtained 9 hot paper, 93 highly cited literatures, and 10 co-cited references. The current research focuses on the following three aspects: EMPA improves left ventricular remodeling, exert renal protection, and increases heart rate variability.

**Conclusion:**

Based on methods such as bibliometrics, citation analysis and knowledge graph, this study analyzed the current situation and trend of EMPA for Heart Failure, sorted out the knowledge context in this field, and provided reference for current and future prevention and scientific research.

## Introduction

Empagliflozin (EMPA), a sodium-glucose cotransporter 2 inhibitor (SGLT2i), is recommended for all patients with Heart failure (HF) who have been treated with ACE-I/ARNI, β-blockers, and MRA, regardless of diabetes, to reduce the risk of Cardiovascular (CV) death, hospitalization, and HF exacerbation in HF patients (1–4). The EMPEROR-Reduced trial found that EMPA reduced the composite primary end point of CV death or HF hospitalization by 25% in patients with NYHA class II-IV symptoms (1) and reduced eGFR decline in individuals. Similarly, EMPA reduced the combined risk of CV death or hospitalization due to HF with a preserved ejection fraction, regardless of diabetes (4). It is also associated with improved quality of life (5, 6). The natriuretic/diuretic properties of EMPA may provide additional benefits regarding reduce congestion and the need for circulatory diuresis (7, 8). In patients with HF with reduced ejection fraction (EF), EMPA reduced the risk and total number of inpatient and outpatient exacerbations of HF events, ameliorate renal endpoints (9), and decrease CV and all-cause death (10), with benefits seen early after treatment initiation and sustained for the duration of double-blind therapy (11).

Changes in cardiac metabolism and ion homeostasis are precursors and drivers of cardiac remodeling and the development of HF. EMPA exerts direct and acute effects on cardiac ion homeostasis by inhibiting the cardiac Na^+^/H^+^ exchanger (NHE) and reducing intracellular Na^+^ and Ca^2+^ (12, 13). Subsequent studies showed for the first time that EMPA had acute specific metabolic effects in isolated type II diabetic db/db mouse hearts, namely, decreased lactate production of labeled glucose and increased α-ketoglutarate synthesis of labeled palmitate, which appeared to be mediated by NHE-1 inhibition (14).

In addition to cardiovascular benefits, EMPA slowed the estimated rate of decline in GFR in double-blind treatment, and the risk of a composite renal outcome was lower in the EMPA group than in the placebo group. Comparing measurements taken at the beginning and end of the trial after discontinuation of EMPA and placebo, the estimated decrease in GFR was greater in the placebo group than in the EMPA group. These observations are consistent with the benefits observed with SGLT2 inhibitors in type 2 diabetic patients who are largely free of HF. Thus, the positive effect of EMPA on renal function in patients with diabetes, HF, and patients with both conditions is evident (1).

Bibliometrics analysis can systematically deal with the original manuscripts of relevant research fields, track current research hotspots and general trends, reveal landmark literatures in this field and obtain clearer and more intuitive results (15–17). CiteSpace and VOSviewer, series of user-friendly bibliometrics analysis software, make the analyzer easy to operate. In this paper, using literature metrology method combing these documents, determine the influential countries, institutions, scholars, journals, and landmark theory, is to summarize the basic situation, provide references for study in the field of EMPA for HF.

## Materials and methods

### Data retrieval and collection

The following category was used to retrieve literature from WOSCC: (Topic = Empagliflozin and Heart Failure). There were no language, publication date, and document type restrictions in the selection of the articles. To avoid data bias, the plain text files containing full record and cited references were exported in the text as download_^***^.txt in under a day (August 10, 2022). In ClinicalTrials.gov^1^, a database of privately and publicly funded clinical studies conducted around the world, we performed a combination search of “Empagliflozin” and “Heart Failure” for clinical trials focused on intervention therapy. Then the retrieval results were analyzed comprehensively according to the stage of the experiment.

### Data analysis

**FIGURE 1:**
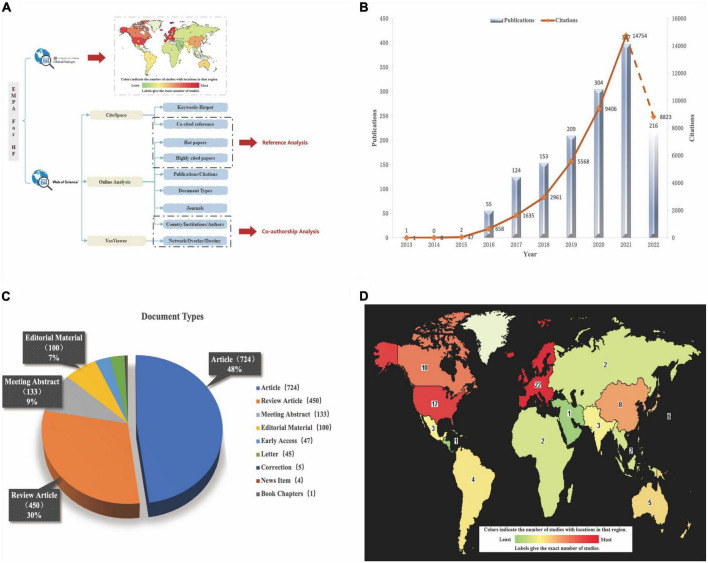
**(A)** The flow diagram of scientometric analysis. **(B)** The trend of Publications and Citations. **(C)** The document types of all literatures. **(D)** Global distribution of clinical trials about EMPA for HF (https://clinicaltrials.gov/ct2/home).

Figure 1A shows a flowchart of the scientometric analysis. We used the WOSCC database for most of the analyses including Publications, Citations, Web of Science Categories, Journal, Country/Region, Affiliations, Authors, hot papers, and highly cited literatures.

VOSviewer (Version 1.6.17) was utilized for the analysis of countries, organizations, and authors. Based on network visualization, VOSviewer divides countries, organizations, or authors into diverse clusters and colors them in the light of the temporal progression of their occurrence, superimposing time on the network of co-occurrence countries, organizations, or authors. The data about country cooperation was converted into GML format by VOSviewer and imported into Scimago Graphica Beta software (Version 1.0.18) to demonstrate geographical distribution and country cluster.

The software CiteSpace (Version 5.8.R3 64-bit) was used for the analysis of co-cited reference and keywords. In the co-occurrence graph, the circle size represents the number of co-citations, and the centrality and burstiness was strikingly marked by the purple and red in the outer ring of the node, respectively. Key points in CiteSpace are nodes with a centrality of more than 0.1. Long-likelihood ration algorithm was used for keywords cluster analysis based on co-occurrence. In the cluster map, the ones surrounded by boxed represent the same cluster. Cluster names are displayed based on the cluster scale.

## Results

### Time distribution map of literatures

It can be seen from Figure 1B that in the decade, the amount of literature published in the field of EMPA for HF showed a rise and peaked in 2021. At present, just 8 months into 2022, the analysis of posts is not included yet. This citation analysis of the 1461 papers on EMPA for HF resulted in a grand total of 43861 times cited in the past 10 years, with a mean of 30.02 citations per paper and h-index of 84. H-index is an important indicator of a researchers’ scientific output (18). The times of citations increased year by year from 1 in 2013 to 14754 in 2021 (Based on the uptrend, citations are likely to continue to break through in 2022). The initial database search yielded 1461 articles, which comprise nine different document types (Figure 1C) including Articles (724 papers, 49.555%), Review Articles (450 papers, 30.801%), Meeting Abstracts (133 papers, 9.103%), Editorial Materials (100 papers, 6.845%), Early Access (47 papers, 3.217%), etc.

### The analysis of clinical trials

To date, EMPA has 42 clinical trials for HF worldwide and the exact distribution is shown in Figure 1D. Of the 42 trials, 21 have been completed, 11 are recruiting patients, 7 have not yet started recruitment, 2 have been terminated by slow enrollment (ClinicalTrials.gov Identifier: NCT03152552) and outbreak involvement (ClinicalTrials.gov Identifier: NCT03554200), and 1 has unknown information (ClinicalTrials.gov Identifier: NCT03271879). Of the 21 completed trials, 10 submitted final findings and all of them had positive effects, as detailed in Table 1. The number of enrolled patients ranged from 23 to 5988, all of whom were over 18 years old and of both sexes. EMPA was used in two specifications: 10 and 25 mg, with 10 mg being the majority and placebo being used as the control drug. The main outcome measures were NT-proBNP, Left Ventricle-End Systolic/Diastolic Volume (LV-EDV/ESV), 6-Min-Walking-Test (6MWT), Kansas City Cardiomyopathy Questionnaire (KCCQ) score, CV death, hospitalization rate, all-cause mortality, and renal function related indicators. The KCCQ, a self-administered questionnaire designed to evaluate physical limitations, symptoms (frequency, severity, and changes over time), social limitations, self-efficacy, and quality of life in patients with HF, incorporates the following domains: symptom burden, symptom frequency, and physical limitation (19). The KCCQ questionnaire has 23 items and 12 items. The KCCQ-12 is a shorter version of the original 23-item test tool, and its credibility has been verified in clinical trials (20).

**TABLE 1:** Completed clinical trials of EMPA in HF.

Identifier	Study title	Interventions	Outcome measures	Populations			Sponsor/Collaborator	Results
				
				Enrollment	Age	Sex		
NCT03030222	Empagliflozin Impact on Hemodynamics in Patients with Heart Failure	Empagliflozin (10 mg)/Placebo	PADP/NT-proBNP/Diuretic Medication Adjustments	65	19–119 Years	All	Saint Luke’s Health System	Positive
NCT03448419	This Study Tests Empagliflozin in Patients with Chronic Heart Failure with Reduced Ejection Fraction (HFrEF). The Study Looks at How Far Patients Can Walk in 6 Minutes and at Their Heart Failure Symptoms	Empagliflozin (10 mg)/Placebo	6MWT Distance/KCCQ (23)-TSS/CHQ-SAS Dyspnea Score/NT-proBNP	312	18 Years and older	All	Boehringer Ingelheim/Eli Lilly and Company	Positive
NCT03448406	This Study Tests Empagliflozin in Patients with Chronic Heart Failure with Preserved Ejection Fraction (HFpEF). The Study Looks at How Far Patients Can Walk in 6 Minutes and at Their Heart Failure Symptoms.	Empagliflozin (10 mg)/Placebo	6MWT Distance/KCCQ (23)-TSS/CHQ-SAS Dyspnea Score/NT-proBNP	315	18 Years and older	All	Boehringer Ingelheim/Eli Lilly and Company	Positive
NCT03057977	Empagliflozin outcome trial in Patients With chronic heart Failure with Reduced Ejection Fraction (EMPEROR-Reduced)	Empagliflozin (10 mg)/Placebo	CV Death or Adjudicated Hospitalization/eGFR (CKD-EPI) cr Slope/renal Endpoint/All-cause Mortality/KCCQ (23) Score	3730	18 Years and older	All	Boehringer Ingelheim/Eli Lilly and Company	Positive
NCT04157751	A Study to Test the Effect of Empagliflozin in Patients Who Are in Hospital for Acute Heart Failure	Empagliflozin (25 mg)/Placebo	NT-proBNP/KCCQ (23)-TSS/CV Death or Adjudicated Hospitalization	530	18 Years and older	All	Boehringer Ingelheim/Eli Lilly and Company	Positive
NCT03057951	Empagliflozin outcome trial in Patients With chronic heart Failure with Preserved Ejection Fraction (EMPEROR-Preserved)	Empagliflozin (10 mg)/Placebo	CV Death or Adjudicated Hospitalization/eGFR (CKD-EPI)/Renal Endpoint/All-cause Mortality/KCCQ (23)	5988	18 Years and older	All	Boehringer Ingelheim/Eli Lilly and Company	Positive
NCT03332212	A Study That Looks at the Function of the Heart in Patients with Heart Failure Who Take Empagliflozin	Empagliflozin (10 mg)/Placebo	ATP Ratio in the Resting State Measured by 31P Cardiac MRS	72	18 Years and older	All	Boehringer Ingelheim/Eli Lilly and Company	Positive
NCT03200860	Effects of Empagliflozin on Clinical Outcomes in Patients with Acute Decompensated Heart Failure	Empagliflozin (10 mg)/Placebo	Dyspnea/Diuretic Response/Length of Stay/NT-proBNP/In hospital Worsening HF all-Cause Mortality Death and/or Heart Failure Re-admission	80	18 Years and older	All	University Medical Center Groningen	Positive
NCT03226457	SGLT2 Inhibition in Combination with Diuretics in Heart Failure	Empagliflozin (25 mg)/Placebo	Urine Output/Urinary Sodium Excretion/Glomerular Filtration Rate/Serum Creatinine/Urinary Protein/Creatinine Ratio/Urinary Albumin/Creatinine Ratio/Renal Biomarker, Cystatin C	23	18 Years and older	All	University of Dundee/British Heart Foundation	Positive
NCT03485222	Are the “Cardiac Benefits” of Empagliflozin Independent of Its Hypoglycemic Activity? (ATRU-4).	Empagliflozin (10 mg)/Placebo	LV-ESV/LV-EDV/EF/VO_2_ Consumption/6MWT/KCCQ-12	84	18 Years and older	All	Icahn School of Medicine at Mount Sinai/Boehringer Ingelheim/Eli Lilly and Company	Positive
NCT03030222	Empagliflozin Impact on Hemodynamics in Patients with Heart Failure	Empagliflozin (10 mg)/Placebo	PADP/NT-proBNP/Diuretic Medication Adjustments	65	19–119 Years	all	Saint Luke’s Health System	Positive

PADP, pulmonary artery diastolic pressure; 6MWT, 6-min-walking- test; KCCQ, Kansas City cardiomyopathy questionnaire; TSS, total symptom score; CHQ-SAS, chronic heart failure questionnaire self-administered standardized format; CV, cardiovascular; MRS, magnetic resonance spectroscopy; FPG, fasting plasma glucose; LV-ESV, left ventricle-end systolic volume; LV-EDV, left ventricle-end diastolic volume.

A series of clinical trials have demonstrated that EMPA improves hemodynamics in patients with HF, rapidly reducing pulmonary artery pressure that is amplified over time and appears to be independent of loop diuresis management (21–23). In the EMPA-REG OUTCOME trial, EMPA reduced CV risk events and influenced mortality and hospitalization in patients with HF independent of its hypoglycemic effect. In addition, the effect of EMPA on exercise capacity in patients with HF did not differ according to LVEF (24–28). In the EMPULSE trial (29), the initiation of EMPA in hospitalized patients with acute HF produced clinical benefits, improved KCCQ scores regardless of the extent of symptom impairment at baseline, and improved symptoms, physical limitations, and quality of life, with benefits seen as early as 15 days and maintained up to 90 days. EMPA resulted in significant reductions in body weight and serum urate, and when combined with loop diuretics, a significant increase in 24-h urine volume but no increase in urinary sodium. It is hypothesized that EMPA’s role as an osmotic diuretic and its effect on natriuretic may underlie the CV and renal benefits (30, 31).

### Web of science disciplines and journals

Table 2 shows the top 15 disciplines. The top three subjects covered by all included literature are Cardiac Cardiovascular Systems (713 papers, 48.802%), Endocrinology Metabolism (352 papers, 24.093%), and Pharmacology Pharmacy (175 papers, 11.978%). The remaining subjects are in order Medicine General Internal (141 papers, 9.651%), Peripheral Vascular Disease (134 papers, 9.172%), Urology Nephrology (54 papers, 3.696%), Medicine Research Experimental (44 papers, 3.012%), Biochemistry Molecular Biology (38 papers, 2.601%), Cell Biology (18 papers, 1.232%), Multidisciplinary Sciences (17 papers, 1.164%), Health Care Sciences Services (16 papers, 1.095%), Toxicology (14 papers, 0.958%), Chemistry Multidisciplinary (12 papers, 0.821%), Chemistry Medicinal (10 papers, 0.684%), and Geriatrics Gerontology (10 papers, 0.684%).

**TABLE 2:** Top 15 disciplines in the field of EMPA for HF in WOSCC.

Rank	Web of science disciplines	Frequency	% of 1461
1	Cardiac cardiovascular systems	713	48.802
2	Endocrinology metabolism	352	24.093
3	Pharmacology pharmacy	175	11.978
4	Medicine general internal	141	9.651
5	Peripheral vascular disease	134	9.172
6	Urology nephrology	54	3.696
7	Medicine research experimental	44	3.012
8	Biochemistry molecular biology	38	2.601
9	Cell biology	18	1.232
10	Multidisciplinary sciences	17	1.164
11	Health care sciences services	16	1.095
12	Toxicology	14	0.958
13	Chemistry multidisciplinary	12	0.821
14	Chemistry medicinal	10	0.684
15	Geriatrics gerontology	10	0.684

Regarding the number of published papers, *Circulation* is the most published journal with 86 articles, with IF of 39.918 and JCR of Q1, followed by *Cardiovascular Diabetology* (71 articles, IF_2021_ = 8.949, Q1), *European Journal Of Heart Failure* (67 articles, IF_2021_ = 17.349, Q1), *Diabetes Obesity Metabolism* (54 articles, IF_2021_ = 6.408, Q1), *European Heart Journal* (39 articles, IF_2021_ = 35.855, Q1), *Journal Of The American College Of Cardiology* (35 articles, IF_2021_ = 27.203, Q1), *Esc Heart Failure* (31 articles, IF_2021_ = 3.612, Q2), *Diabetologia* (27 articles, IF_2021_ = 10.46, Q1), *Heart Failure Reviews* (25 articles, IF_2021_ = 4.654, Q2) and *New England Journal Of Medicine* (24 articles, IF_2021_ = 176.079, Q1) (Table 3).

**TABLE 3:** Top 15 contributing journals in the field of EMPA for HF.

Rank	Journal	Frequency	% of 1461	IF_2021_	JCR	Average per item	H-index
1	Circulation	86	5.886	39.918	Q1	54.6	28
2	Cardiovascular Diabetology	71	4.86	8.949	Q1	25.65	26
3	European Journal of Heart Failure	67	4.586	17.349	Q1	27.52	20
4	Diabetes Obesity Metabolism	54	3.696	6.408	Q1	23.52	19
5	European Heart Journal	39	2.669	35.855	Q1	30.59	11
6	Journal of the American College of Cardiology	35	2.396	27.203	Q1	44.29	15
7	Esc Heart Failure	31	2.122	3.612	Q2	10.97	9
8	Diabetologia	27	1.848	10.46	Q1	51.59	10
9	Heart Failure Reviews	25	1.711	4.654	Q2	9.32	9
10	New England Journal of Medicine	24	1.643	176.079	Q1	639.58	11
11	Journal of the American Heart Association	21	1.437	6.016	Q2	18.33	11
12	Diabetes Therapy	19	1.3	3.595	Q3	9.79	8
13	Cardiovascular Drugs and Therapy	16	1.095	3.947	Q2	15.31	8
14	Diabetes Research and Clinical Practice	16	1.095	8.18	Q1	7.81	7
15	Diabetes Care	15	1.027	17.152	Q1	76.67	13

### The analysis of co-authorship–Countries/regions, research institutions and influential scholars

**FIGURE 2:**
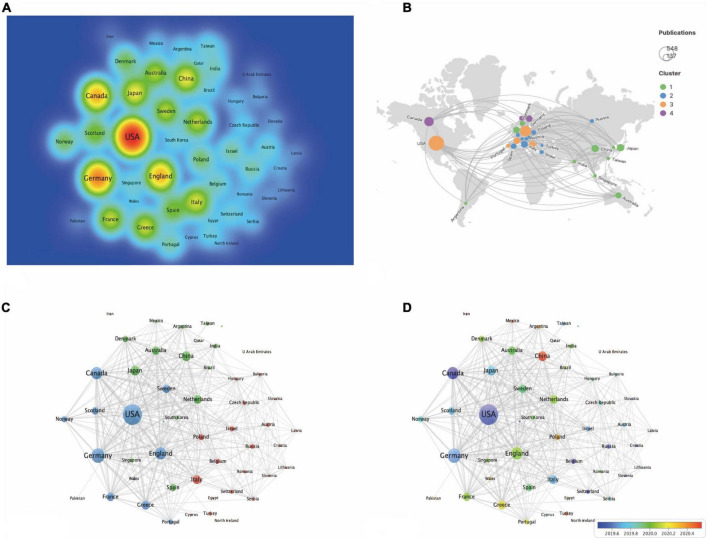
The analysis of country/region. **(A)** The destiny of all contributing countries. **(B)** The cluster of all contributing countries by Scimago. **(C)** The occurrence of all contributing countries. **(D)** The time overlay of all contributing countries.

In terms of publication numbers, USA is the country with the highest number of publications, accounting for 37 percent of the total, followed by the Germany (286 papers, 19.576%), Canada (213 papers, 14.579%), England (208 papers, 14.237%), Japan (135 papers, 9.24%), China (133 papers, 9.103%), Italy (121 papers, 8.282%), Greece (114 papers, 7.803%), France (110 papers, 7.529%), and Netherlands (107 papers, 7.324%) (Table 4 and Figure 2A). Figure 2B shows geographical distribution of all contributing countries. Of these top 15 countries/regions, ten from Europe, two are from Asia, two from North America and one from Oceania. The total citations, total link strength and h-index ranked first in the USA, which were 28,404, 1,194, and 70 respectively, suggesting that the USA is relatively mature in the field of EMPA for HF. As shown in Figure 2C, 51 countries met the threshold, and 3 clusters were generated, among which the largest clusters were 21 countries, which are represented by red, green, and blue. In Figure 2D, the more purple the color, the earlier the study, and the redder the later the study. It shows that the USA, Canada, and Germany were studied earlier. China, Mexico, and Turkey appear later, around 2021.

**TABLE 4:** Top 15 contributing countries/regions in the field of EMPA for HF.

Rank	Countries/Regions	Frequency	% of 1461	Average per item	H-index	Citations	Total link strength
1	USA	555	37.988	56.98	70	28404	1194
2	Germany	286	19.576	60.2	44	16290	937
3	Canada	213	14.579	110.89	47	22678	714
4	England	208	14.237	103.48	48	20587	857
5	Japan	135	9.24	123.46	34	16226	259
6	China	133	9.103	110.5	31	14347	262
7	Italy	121	8.282	86.75	32	10,203	341
8	Greece	114	7.803	90.73	32	10,025	452
9	France	110	7.529	112.26	36	12,040	478
10	Netherlands	107	7.324	166.42	39	17,406	529
11	Australia	99	6.776	135.75	30	13,168	376
12	Sweden	97	6.639	117.84	37	11,128	527
13	Scotland	83	5.681	167.76	35	13,647	393
14	Spain	79	5.407	111.29	25	8,668	197
15	Denmark	66	4.517	180.02	30	11,687	278

**FIGURE 3:**
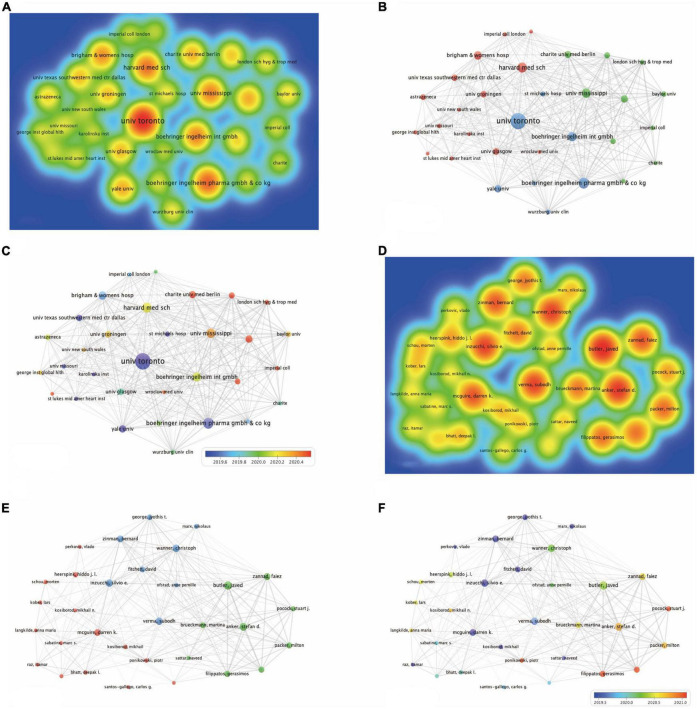
The analysis of institutions and authors. **(A)** The destiny of all contributing institutions. **(B)** The occurrence of all contributing institutions. **(C)** The time overlay of all contributing institutions. **(D)** The destiny of all contributing authors. **(E)** The occurrence of all contributing authors. **(F)** The time overlay of all contributing authors.

The three institutions with the largest number of publications were from University of Toronto, Boehringer Ingelheim and Saint Michaels Hospital Toronto, accounting for 11.567, 10.678, and 8.966% of the total reports respectively, will in Table 5 and Figure 3A. Six of the top 15 published research institutions are in the USA, five are in Germany, while two are in Canada. University of Toronto is the research institution with the most total citations, with an API of 134.19 and an h-index of 44, followed by Harvard University and Saint Michaels Hospital Toronto. Figure 3B shows the connections between institutions. Nodes represent the number of publications issued by institutions, and lines represent the connections between institutions. Three clusters are formed with red, blue, and green respectively. As can be seen from the time superposition chart of institutions (Figure 3C), the study of University of Toronto started earlier, while Charite Universitatsmedizin Berlin appeared later, around the end of 2020.

**TABLE 5:** Top 15 contributing institutions in the field of EMPA for HF.

Rank	Institutions	Frequency	% of 1461	Average per item	H-index	Times cited	Countries
1	University of Toronto	169	11.567	134.19	44	21,923	Canada
2	Boehringer Ingelheim	156	10.678	66.17	32	9,991	Germany
3	Saint Michaels Hospital Toronto	131	8.966	104.18	36	13,202	Canada
4	Harvard University	126	8.624	124.48	41	15,123	USA
5	Brigham Women S Hospital	96	6.571	140.1	36	13,045	USA
6	Charite Universitatsmedizin Berlin	90	6.16	46.06	26	3,938	Germany
7	Free University of Berlin	90	6.16	46.06	26	3,938	Germany
8	Humboldt University of Berlin	90	6.16	46.06	26	3,938	Germany
9	Harvard Medical School	87	5.955	112.8	34	9,539	USA
10	University of Wurzburg	83	5.681	97.28	22	7,934	Germany
11	University of Texas System	80	5.476	114.63	30	8,975	USA
12	Yale University	78	5.339	138.65	28	10,514	USA
13	University of Mississippi	76	5.202	47.13	25	3,406	USA
14	National Kapodistrian University of Athens	75	5.133	54.08	25	3,874	Greece
15	University of London	71	4.86	89.04	23	6,135	England

With regards to authors, Butler J from the University of Mississippi was the most prolific, followed by Anker SD from Charite Universitatsmedizin Berlin and Inzucchi SE from Yale University. Of the top 15 authors, eight are from the USA and Germany, evenly split. Inzucchi SE from Yale University have the most citations of 1,0007 and the highest h-index of 26 on the field of EMPA for HF (Table 6 and Figure 3D). Figure 3E shows the connections between authors. Nodes represent the number of publications issued by authors, and lines represent the connections between authors. Three clusters are formed with red, blue, and green respectively. The clustering formed by Zinman B, Fitchett D, and Mcguire DK appeared earliest. Since then, many scholars have conducted in-depth research (Figure 3F).

**TABLE 6:** Top 15 contributing authors in the field of EMPA for HF.

Rank	Authors	Frequency	% of 1461	Average per item	*H*-index	Times cited	Affiliations	Country
1	Butler J	81	5.544	50.57	26	3,890	University of Mississippi	USA
2	Anker SD	78	5.339	50.5	24	3,752	Charite Universitatsmedizin Berlin	Germany
3	Inzucchi SE	73	4.997	138.93	26	10,007	Yale University	USA
4	Wanner C	71	4.86	112.25	20	7,845	University of Wurzburg	Germany
5	Zinman B	66	4.517	135.95	21	8,864	Mt Sinai Hospital	Canada
6	Verma S	63	4.312	97.48	24	5,995	University of Toronto	Canada
7	Brueckmann M	56	3.833	58.45	23	3,175	Boehringer Ingelheim	Germany
8	Fitchett D	56	3.833	116.8	17	6,466	University of Toronto	Canada
9	Zannad F	55	3.765	62.38	21	3,308	University of Lorraine	France
10	Mcguire DK	51	3.491	183.65	25	9,223	University of Texas System	USA
11	Filippatos G	49	3.354	67.12	20	3,185	National Kapodistrian University of Athens	Greece
12	George JT	48	3.285	20.98	17	980	Boehringer Ingelheim	Germany
13	Packer M	47	3.217	68.51	18	3,107	Baylor University	USA
14	Ferreira JP	43	2.943	37.93	17	1,560	University of Lorraine	France
15	Ponikowski P	38	2.601	145.66	21	5,437	Wrocław Medical University	Poland

### The analysis of reference—Hot papers, highly cited literatures, and co-cited reference

#### Hot papers

Hot papers were those published in the past 2 years and received enough citations in November/December 2021 to be in the top 0.1% of papers in an academic field in a subject area. There were nine hot papers (1, 4, 10, 32–37) in total, and all of them were highly cited (Table 7). The types of documents are all articles, and all of them are published in the journal with JCR of Q1, most of which were *New England Journal of Medicine* (IF_2021_ = 176.079, Q1). The above nine hot papers included two meta-analyses, and the results showed that SGLT2 inhibitors were associated with a reduced risk of major adverse CV events and renal outcomes (36), as well as a reduced combined risk of CV death or hospitalization due to HF in patients with or without diabetes (4). The results were confirmed in another study (10). Three other trials (1, 32, 35), all of which looked at the effects of SGLT2i (EMPA, dapagliflozin, and sotagliflozin) on the kidneys, were also positive.

**TABLE 7:** The total nine hot papers in the field of EMPA for HF.

Article title	Document type	Citations	Journals	DOI	Highly cited
Cardiovascular and Renal Outcomes with Empagliflozin in Heart Failure	Article	1199	New Engl J Med	10.1056/NEJMoa2022190	Yes
Dapagliflozin in Patients with Chronic Kidney Disease	Article	977	New Engl J Med	10.1056/NEJMoa2024816	Yes
Empagliflozin in Heart Failure with a Preserved Ejection Fraction	Article	476	New Engl J Med	10.1056/NEJMoa2107038	Yes
Sotagliflozin in Patients with Diabetes and Recent Worsening Heart Failure	Article	462	New Engl J Med	10.1056/NEJMoa2030183	Yes
Cardiovascular Outcomes with Ertugliflozin in Type 2 Diabetes	Article	456	New Engl J Med	10.1056/NEJMoa2004967	Yes
SGLT2 inhibitors in patients with heart failure with reduced ejection fraction: a meta-analysis of the EMPEROR-Reduced and DAPA-HF trials	Article	414	Lancet	10.1016/S0140-6736(20)31824-9	Yes
Sotagliflozin in Patients with Diabetes and Chronic Kidney Disease	Article	282	New Engl J Med	10.1056/NEJMoa2030186	Yes
Association of SGLT2 Inhibitors with Cardiovascular and Kidney Outcomes in Patients With Type 2 Diabetes A Meta-analysis	Article	221	Jama Cardiol	10.1001/jamacardio.2020.4511	Yes
Effect of Empagliflozin on Worsening Heart Failure Events in Patients with Heart Failure and Preserved Ejection Fraction EMPEROR-Preserved Trial	Article	42	Circulation	10.1161/CIRCULATIONAHA.121.056824	Yes

#### Highly cited literatures

As of November/December 2021, these highly cited literatures received enough citations to place it in the top 1% of the academic field based on a highly cited threshold for the field and publication year. A total of 93 highly cited literatures were detected in the database in this research field, with the highest citation frequency of 4753. Among them, the above nine hot papers were all among the highly cited articles. Therefore, the top 15 highly cited literatures excluding the hot papers are shown in Table 8. With regards to the document type, 12 were articles and 3 were review. The publication time of highly cited literatures was generally from 2015 to 2019. Among the top 15 highly cited literatures, only one described the underlying mechanism of EMPA in the treatment of HF, involving *in vivo* experiments in animals (12). Elevated cardiac cytoplasmic Na^+^([Na^+^]_*c*_) and Ca^2+^([Ca^2+^]_*c*_) concentrations and decreased mitochondrial Ca^2+^([Ca^2+^]_*m*_) concentrations are driving factors for HF and cardiac death (38, 39). Previous studies (40, 41) have shown that chronic NHE inhibition prevents or attenuates HF in animal models. Ventricular myocytes of rabbits and rats were acutely isolated, and the activities of [Na^+^]_*c*_, [Ca^2+^]_*c*_, [Ca^2+^]_*m*_ and NHE were measured by fluorimetric assay. The results showed that EMPA had a direct effect on the heart by injuring myocardial NHE flux, reducing myocardial [Na^+^]_*c*_ and [Ca^2+^]_*c*_, and enhancing [Ca^2+^]_*m*_, independent of SGLT2 activity (12). Thus, it is hypothesized that the beneficial CV effects of EMPA are at least partially attributable to NHE inhibition, and it is appropriate to complement the mechanistic studies of EMPA in the treatment of HF.

**TABLE 8:** The top 15 highly cited literatures in the field of EMPA for HF.

Article title	Document type	Citations	Journal	Year	DOI
Empagliflozin, Cardiovascular Outcomes, and Mortality in Type 2 Diabetes	Article	4753	New Engl J Med	2015	10.1056/NEJMoa1504720
Dapagliflozin and Cardiovascular Outcomes in Type 2 Diabetes	Article	2515	New Engl J Med	2019	10.1056/NEJMoa1812389
Dapagliflozin in Patients with Heart Failure and Reduced Ejection Fraction	Article	2130	New Engl J Med	2019	10.1056/NEJMoa1911303
Canagliflozin and Renal Outcomes in Type 2 Diabetes and Nephropathy	Article	2071	New Engl J Med	2019	10.1056/NEJMoa1811744
SGLT2 inhibitors for primary and secondary prevention of cardiovascular and renal outcomes in type 2 diabetes: a systematic review and meta-analysis of cardiovascular outcome trials	Review	1283	Lancet	2019	10.1016/S0140-6736(18)32590-X
Heart failure outcomes with empagliflozin in patients with type 2 diabetes at high cardiovascular risk: results of the EMPA-REG OUTCOME (R) trial	Article	640	Eur Heart J	2016	10.1093/eurheartj/ehv728
Sodium Glucose Cotransporter 2 Inhibitors in the Treatment of Diabetes Mellitus: Cardiovascular and Kidney Effects, Potential Mechanisms, and Clinical Applications	Article	639	Circulation	2016	10.1161/CIRCULATIONAHA.116.021887
Lower Risk of Heart Failure and Death in Patients Initiated on Sodium-Glucose Cotransporter-2 Inhibitors Versus Other Glucose-Lowering Drugs The CVD-REAL Study (Comparative Effectiveness of Cardiovascular Outcomes in New Users of Sodium-Glucose Cotransporter-2 Inhibitors)	Article	523	Circulation	2017	10.1161/CIRCULATIONAHA.117.029190
SGLT2 inhibitors and mechanisms of cardiovascular benefit: a state-of-the-art review	Review	388	Diabetologia	2018	10.1007/s00125-018-4670-7
Can a Shift in Fuel Energetics Explain the Beneficial Cardiorenal Outcomes in the EMPA-REG OUTCOME Study? A Unifying Hypothesis	Article	372	Diabetes Care	2016	10.2337/dc16-0542
Comparison of the Effects of Glucagon-Like Peptide Receptor Agonists and Sodium-Glucose Cotransporter 2 Inhibitors for Prevention of Major Adverse Cardiovascular and Renal Outcomes in Type 2 Diabetes Mellitus Systematic Review and Meta-Analysis of Cardiovascular Outcomes Trials	Review	349	Circulation	2019	10.1161/CIRCULATIONAHA.118.038868
Empagliflozin decreases myocardial cytoplasmic Na + through inhibition of the cardiac Na + /H + exchanger in rats and rabbits	Article	325	Diabetologia	2017	10.1007/s00125-016-4134-x
Effect of Dapagliflozin on Heart Failure and Mortality in Type 2 Diabetes Mellitus	Article	298	Circulation	2019	10.1161/CIRCULATIONAHA.119.040130
Effects of sodium-glucose cotransporter-2 inhibitors on cardiovascular events, death, and major safety outcomes in adults with type 2 diabetes: a systematic review and meta-analysis	Article	290	Lancet Diabetes Endo	2016	10.1016/S2213-8587(16)00052-8
Type 2 diabetes mellitus and heart failure: a position statement from the Heart Failure Association of the European Society of Cardiology	Article	286	Eur J Heart Fail	2018	10.1002/ejhf.1170

#### Co-cited references

**FIGURE 4:**
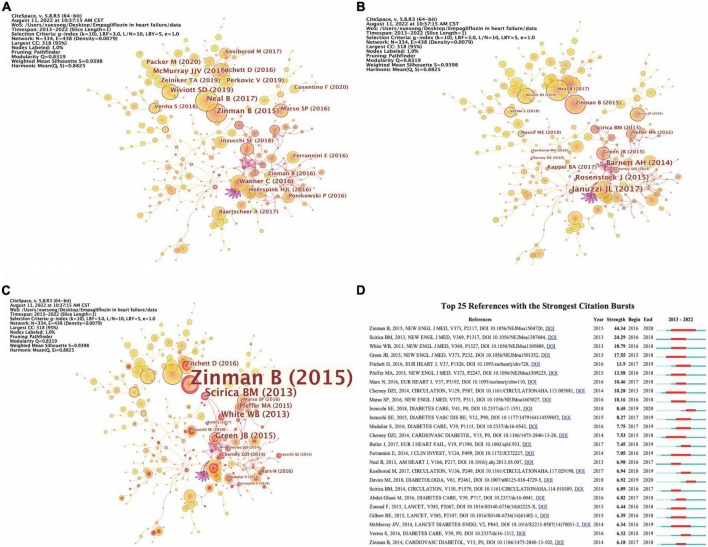
The analysis of co-cited references. **(A)** The occurrence of co-cited references by citations. **(B)** The occurrence of co-cited references by centrality. **(C)** The occurrence of co-cited references by burst intensity. **(D)** The emergence of co-cited references.

The analysis of co-cited references reveals that there are two references in the list of the third citation article, and then the two references form a co-citation relationship. Figures 4A–D respectively represents the citations, centrality, and burst intensity of co-cited literature. The blue line in the figure represents the time axis of the existence of included literatures, and the red line represents the period of the strongest emergence of corresponding literatures. In the co-occurrence diagram of co-cited literatures, the line between nodes means that two literatures appear in the reference of the same article. The more the line is, the articles have certain similarity in content. Nodes represent the citations of literature, purple outside nodes represents the centrality of literature, and red represents the intensity of highlighting. According to the intensity of burst, the top 10 literatures are shown in Table 9.

**TABLE 9:** The 10 co-cited references with burst intensity in the field of EMPA for HF.

Title	References	Journal	Burst intensity	Frequency	Centrality	DOI
Empagliflozin, Cardiovascular Outcomes, and Mortality in Type 2 Diabetes	Zinman et al. (42)	New Engl J Med	44.34	334	0.37	10.1056/NEJMoa1504720
Saxagliptin and cardiovascular outcomes in patients with type 2 diabetes mellitus	Scirica et al. (108)	New Engl J Med	24.29	49	0.35	10.1056/NEJMoa1307684
Alogliptin after acute coronary syndrome in patients with type 2 diabetes	White et al. (109)	New Engl J Med	18.79	38	0.19	10.1056/NEJMoa1305889
Saxagliptin and cardiovascular outcomes in patients with type 2 diabetes mellitus	Green et al. (110)	New Engl J Med	17.55	68	0.33	10.1056/NEJMoa1501352
Heart failure outcomes with empagliflozin in patients with type 2 diabetes at high cardiovascular risk: results of the EMPA-REG OUTCOME^®^ trial	Fitchett et al. (24)	Eur Heart J	13.9	151	0.13	10.1093/eurheartj/ehv728
Lixisenatide in Patients with Type 2 Diabetes and Acute Coronary Syndrome	Pfeffer et al. (111)	New Engl J Med	13.58	50	0.3	10.1056/NEJMoa1509225
Sodium-glucose cotransporter-2 inhibition for the reduction of cardiovascular events in high-risk patients with diabetes mellitus	Marx et al. (112)	Eur Heart J	10.46	27	0.01	10.1093/eurheartj/ehw110
Renal hemodynamic effect of sodium-glucose cotransporter 2 inhibition in patients with type 1 diabetes mellitus	Cherney et al. (102)	Circulation	10.28	28	0.16	10.1161/CIRCULATIONAHA.113.005081
Liraglutide and Cardiovascular Outcomes in Type 2 Diabetes	Marso et al. (113)	New Engl J Med	10.16	91	0.2	10.1056/NEJMoa1603827
Empagliflozin and Assessment of Lower-Limb Amputations in the EMPA-REG OUTCOME Trial	Inzucchi et al. (25)	Diabetes Care	8.45	72	0	10.2337/dc17-1551

The first article named “Empagliflozin, Cardiovascular Outcomes, and Mortality in Type 2 Diabetes,” which written by Zinman B and published in the *New England Journal of Medicine* (IF_2021_ = 176.079) in 2015, is the first study to provide evidence that antidiabetic agents reduce CV events (42). In the EMPA-REG OUTCOME trials, a total of 7020 patients with T2DM were randomized to receive either 10 or 25 mg of EMPA daily or placebo. Compared with placebo, EMPA significantly reduced the risk of death from CV causes, non-fatal myocardial infarction, or non-fatal stroke. The results were surprising; In contrast to other CV risk reducing interventions, such as lowering LDL-C (43) and Blood Pressure (44), CV death was significantly reduced by 38% (*P* = 0.001). The separation of Kaplan–Meier curves for CV mortality and HF hospitalization observed during the first 3 months of the trial suggests that the treatment mechanism for EMPA has an early and profound effect on the risk of death or HF (42). Another article titled “Heart Failure Outcomes with Empagliflozin in patients with Type 2 Diabetes at High Cardiovascular Risk: Results of the EMPA-Reg OUTCOME^®^ Trial,” written by Fitchett D, published in the *European Heart Journal* (IF_2021_ = 35.855) in 2016, highlighting the strength 13.9, also reported these findings from the same randomized controlled trial (ClinicalTrials.gov Identifier: NCT01131676).

### Keywords analysis and visualization

**FIGURE 5:**
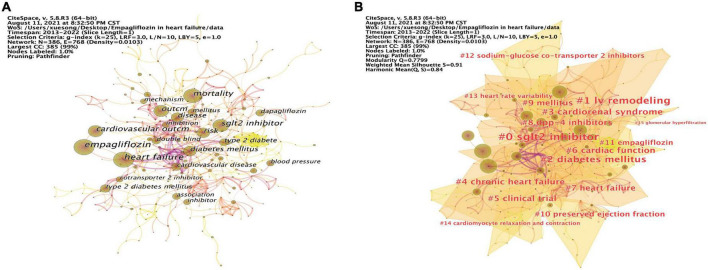
Keywords analysis and visualization. **(A)** The occurrence of keywords. **(B)** The cluster of keywords.

Our analysis of the co-occurrence diagram was used to see which were the main directions and hotspots of EMPA for HF research, as well as to understand how its themes and topics developed and changed over time. The keyword co-occurrence diagram of 386 nodes and 768 links was obtained by running CiteSpace (Figure 5A). The node size represents the frequency of keywords. The centrality of keywords was calculated by “Node-compute Node Centrality” in CiteSpace, and the top 10 keywords in frequency and centrality were summarized.

As previous studies (45), based on keyword co-occurrence graph, the keyword clustering analysis was carried out (Figure 5B). The evaluation diagram was mainly judged from the clustering effect and reliability. Modularity *Q* = 0.7799, the closer *Q* value was to 1, the better the clustering result of the network was, and *Q* value > 0.3 indicated that the clustering structure was significant. Silhouette *S* = 0.91, which measured network homogeneity. When Silhouette was more closely to 1, the more homogenous the Silhouette would be. An *S* value > 0.5 was considered reasonable and an *S* value > 0.7 was considered convincing (46). The cluster information is exported in “Summary Table/Whitelists” on the cluster menu bar of CiteSpace, and the key information was displayed in Table 10. A total of 16 clusters were formed in this study. The smaller the number, the larger the cluster size was. #0 sglt2 inhibitor was the largest cluster, followed by #1 lv remodeling, #2 diabetes mellitus, #3 cardiorenal syndrome, #4 chronic heart failure, #5 clinical trial, #6 cardiac function, #7 heart failure, #8 dpp-4 inhibitors, #9 mellitus, #10 preserved ejection fraction, #11 empagliflozin, #12 sodium-glucose co-transporter 2 inhibitors, #13 heart rate variability, #14 cardiomyocyte relaxation and contraction, and #15 glomerular hyperfiltration.

**TABLE 10:** The total 16 clusters of keywords.

Cluster	Size	Silhouette	Year	LLR
0	40	0.933	2018	sglt2 inhibitor
1	36	0.924	2018	lv remodeling
2	32	0.938	2016	Diabetes mellitus
3	28	0.834	2018	Cardiorenal syndrome
4	27	0.861	2017	Chronic heart failure
5	27	0.838	2020	Clinical trial
6	27	0.922	2019	Cardiac function
7	25	0.97	2017	Heart failure
8	25	0.853	2020	dpp-4 inhibitors
9	25	0.864	2017	Mellitus
10	21	0.945	2018	Preserved ejection fraction
11	20	0.99	2020	Empagliflozin
12	18	0.953	2019	Sodium-glucose co-transporter 2 inhibitors
13	15	0.882	2018	Heart rate variability
14	12	0.956	2019	Cardiomyocyte relaxation and contraction
15	7	0.993	2018	Glomerular hyperfiltration

## Discussion

Different from traditional review, bibliometrics analysis can systematically deal with the original manuscripts of relevant research fields, track current research hotspots and general trends, reveal landmark literatures in this field, and obtain clearer and more intuitive results. CiteSpace and VOSviewer, series of user-friendly bibliometrics analysis software, make the analyzer easy to operate. In addition, we use other graphics software, such as Scimago Graphica, to make the data more visible.

The WOSCC system is the most famous database of scientific publications regarding many investigations. According to the literature screening strategy mentioned earlier, 1461 literatures about EMPA for HF in the decade were extracted from WOSCC, with a total of 43861 references, and the number of manuscripts was on a rise. Compared with the h-index, the mean number of citations per paper is a superior indicator of scientific quality, in terms of both accuracy and precision (47). As shown in the geographical distribution map of all contributing countries, most of the research comes from developed countries, and the Americas, Asia and Europe are heavily published and closely linked. Africa has few publications and few contacts with other countries, which suggests that countries should further break regional barriers for academic exchanges.

In the WOSCC database, there are 45 disciplines involved in the field of EMPA for HF and the top 15 are summarized in Table 2. Among them, multidisciplinary research shows an outbreak trend in the current research stage, which is conducive to future development. Most of the JCR in the top 15 journals are Q1/Q2, and seven articles had an IF (an index of the quality of articles included in the journal) of more than 10, with the highest score of 176.079, and four of them scored 5–10, suggesting that the quality of articles written by scholars in this field is high and can provide better guidance for new researchers.

Based on keywords co-occurrence, clustering and burstiness studies, the current research focuses on the following three aspects: EMPA improves left ventricular remodeling, exert renal protection, and increases heart rate variability (HRV).

### Empagliflozin improves left ventricular remodeling

Acute or chronic myocardial injury results in metabolic disorders followed by cardiomyocyte death. The remaining viable myocardium copes with cell loss through the development of cardiomyocyte hypertrophy and fibrosis in the interstitial space (48, 49). A reduction in contractile force activates neurohormonal compensatory mechanisms designed to maintain systemic perfusion, such as the sympathetic and renal angiotensin systems. Structural changes in the left ventricular (LV) occurred over time, including an increase in ventricular volume, a shift to the right of the end-diastolic pressure-volume relationship (EDPVR), and a development of sphericity (50–52). These anatomical alterations are accompanied by alterations in gene expression, metabolic substrate preference and utilization, and extracellular matrix composition (53–55). These structural, biochemical, and molecular changes are collectively known as ventricular remodeling.

Ventricular remodeling, first described in animal models of LV stress and injury, occurs progressively after large myocardial infarction and in patients with dilated cardiomyopathy. Ventricular remodeling refers to changes in ventricular structure, associated with increased volume and changes in ventricular structure, which are driven by pathological cardiomyocyte hypertrophy, cardiomyocyte apoptosis, myofibroblast proliferation, and interstitial fibrosis at the histological level (56–58). LVEF, the most used clinical measure of cardiac function, is more affected by the degree of LV remodeling than any other factor. Other more precise remodeling measures, such as LV volume and mass, have received greater attention in clinical trials than in clinical practice, but these measures are more closely associated with prognostic and therapeutic effects than LVEF (59). Numerous studies have shown that LV volume measurements at a single time point and over time are of great value in predicting clinical outcomes in patients with HF. A recent meta-analysis of HF trials showed that LV volume stood out among surrogate measures and was strongly associated with the impact of specific drug or device treatment on patient survival. These findings confirm the importance of ventricular remodeling in advancing the pathophysiology of HF and support the role of ventricular remodeling measures in clinical studies of new treatments for HF (60).

EMPATROPISM trial, a pioneer trial to be published first, demonstrated a reduction in LV volumes, regression in LV mass, improvement in LVEF, amelioration in LV sphericity (61). Moreover, EMPA also improved peak oxygen consumption (62), the gold-standard parameter evaluating exercise capacity. EMPA also reduced LV interstitial fibrosis and aortic stiffness (63). The pioneering results of EMPATROPISM have been subsequently confirmed by other independent trials such as SUGAR-DM (64) and eMPIRE-HF (62). In the EMPA-HEART trial (61), the mean LV mass in the regression of body surface area after 6 months was 2.6 g/m^2^ in the EMPA group and 0.01 g/m^2^ in the placebo group (*P* = 0.01), with a significant reduction in the EMPA group, which is presumed to have a positive effect on CV outcomes.

The results were verified in animal experiments. Researchers used LAD ligation to establish a model of HF after MI in female Yorkshire pigs and randomly assigned them to either EMPA or placebo for 2 months. Myocardial injury was subsequently detected using cardiac magnetic resonance and three-dimensional (3D) Echocardiography. The LV end-systolic and end-diastolic diameters of EMPA treated pigs were significantly reduced, indicating a significant mitigation in LV dilatation. LV mass was significantly reduced in EMPA treated pigs and the degree of sphericity of the heart was lower than in control pigs, as indicated by the lower 3D-LV sphericity index, thus indicating reduced structural changes in the treated arm. Further CMR results showed that the improvement of LV remodeling in the EMPA group was associated with an increase in LVEF 2 months after treatment (65). Santos-Gallego demonstrated that the improvement in LVEF is due to a switch in myocardial metabolism. EMPA induces a shift in myocardial metabolism away from glucose-inefficient glucose toward the consumption of free fatty acid and ketone bodies, which enhances cardiac energetics (66, 67). The circumference of porcine cardiomyocytes treated with EMPA was smaller than that in the control group, indicating that there was less compensatory hypertrophy at the cellular level in the EMPA group (68). Increased interstitial myocardial fibrosis (IMF) causes diastolic dysfunction. Collagen deposition was reduced in EMPA-treated pigs versus controls, and levels of hydroxyproline, an essential amino acid found only in collagen and thus an indirect marker of IMF, were lower in the EMPA group. IMF was reduced as the treated group exhibited stiffer Type-1 collagen and reduced expression of the pro-fibrotic cytokine TGF-B gene, as well as reduced TGF-B activity (68). This suggests that EMPA can ameliorate IMF, thereby improving ventricular remodeling.

### Reno protective effects of empagliflozin

In the EMPEROR-Reduced Trial, EMPA slowed the progression of eGFR reduction compared with placebo (69, 70). EMPA resulted in a similar placebo-adjusted reduction in the urinary albumin-to-creatinine ratio (UACR) over a mean period of 3.1 years, regardless of Obstructive Sleep Apnea status at baseline (71). EMPA has moderate diuretic and natriuretic effects that ameliorate OSA symptoms by reducing fluid retention in the pleural cavity (72) and rostral fluid transfer to the legs (73), as well as affecting kidney sodium handling (74). Moreover, EMPA induces natriuresis, which enhances the diuretic effect of loop diuretics (8), but does seems only to be maintained in the acute phase (30). It is this acute natriuretic effect which explains the quick benefits of EMPA in acute heart failure within the first days (75).

Brenner (76) proposed that glomerular hyperfiltration with glomerular capillary hypertension is the pathogenesis of renal disease, among which glomerular hyperfiltration has been considered as a potential risk factor for Diabetic nephropathy (DN) (77). SGLT2i reduce glomerular hyperfiltration, thereby preventing the progression of DN, which may in turn reduce CV risk, including HF. Although EMPA have shown significant renal and CV benefits in humans in clinical trials (42, 78), the mechanism of action is not fully understood but is attributable, at least in part, to a reduction in intraglomerular hypertension and hyperfiltration. Single-nephron GFR (SNGFR) in spontaneously diabetic *Ins2*^+/Akita^ mice was significantly higher than in control groups, reflecting glomerular hyperfiltration, which was ameliorated in EMPA treatment (79).

Hyperuricemia is common in HF and is an independent predictor of advanced disease severity and increased mortality. In the EMPEROR-reduced trial, EMPA induced a rapid and sustained reduction of serum uric acid levels and of clinical events related to hyperuricemia and gout (80). Diabetes mellitus is a risk factor for nephrolithiasis. In an observational study involving 24,290 individuals with T2DM taking SGLT2 inhibitors, Kristensen et al. reported that SGLT2 inhibitor use was associated with a 49% lower risk of nephrolithiasis compared with GLP-1 receptor agonists (81). In EMPA-REG OUTCOME trial, compared with placebo, EMPA therapy was associated with an approximate 40% reduced risk of urinary tract stone events. The underlying mechanisms are unknown but may involve altered lithogenic profile of the urine (82).

Progressive decline in renal function often leads to chronic cardiac dysfunction and CV events. HF is a common complication in patients with chronic kidney disease (CKD), and this process is defined as type 4 cardiorenal syndrome. Trials have shown that SGLT2i treatment significantly improves the outcome of HF, reducing the length of hospitalization for this complication in CKD patients by 30–40% (83). Indeed, treatment with EMPA in diabetic adipose rats improved systemic endothelial function (84). These effects are thought to be mediated probably through increased endothelial viability, reduced senescence, and inflammation, and reduced oxidative damage in the nitric oxide (NO)/cycloguanosine monophosphate signaling cascade, rather than upregulation of endothelial NO synthase expression (84).

**FIGURE 6:**
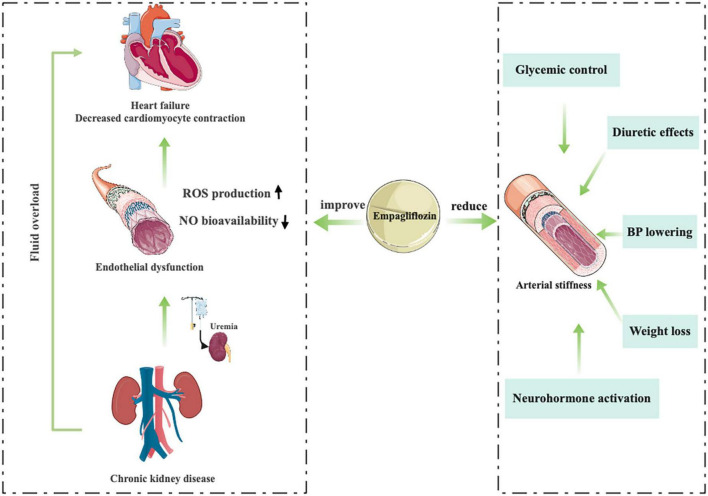
Empagliflozin improves endothelial function and reduces arterial stiffness.

Juni et al. (85) provide an interesting mechanistic insight into how uremia impinges on the interaction between endothelial cells and cardiomyocytes and highlight the critical role of cardiac microvascular endothelium in the cardiac benefits of EMPA treatment (Figure 6). The researchers used a co-culture model of human cardiac microvascular endothelial cells (CMECs) and rat ventricular cardiomyocytes. The results showed that CMECs positively affected the relaxation and contraction of cardiomyocytes mainly through endoderm-derived NO. They demonstrated that uremic serum impairs endothelium mediated enhancement of cardiomyocyte function and that EMPA counteracts the deleterious effects of uremic serum on CMECs, leading to restoration of cardiomyocyte function (86).

Arterial stiffness, a surrogate marker of renal and CV clinical outcomes, is influenced by activation of the renin angiotensin aldosterone system (RAAS) and sympathetic nervous system (SNS) and inhibition of NO (87, 88), increases under the influence of ambient hyperglycemia and can be ameliorated by strict glycemic control (89–91), which may help improve BP control and reduce the risk of CV complications (92–94). Traditional oral hypoglycemic agents (liraglutide or glibenclamide) do not improve arterial stiffness parameters, and a significant proportion of patients develop hypertension as the duration of diabetes increases (95, 96). In an 8-week, open-label, prospective clinical trial (NCT01392560) (97), the novel oral hypoglycemic agents, EMPA, could significantly reduce the parameters of vascular sclerosis, including radial augmentation Index, various sclerosis Index and aortic augmentation index. They suggest that the physiological mechanism of reduced arterial stiffness may be related to a variety of factors, including BP lowering, diuretic effects, altered neurohormone activation, improved glycemic control, and weight loss (Figure 6) (98–103).

### Empagliflozin increases heart rate variability

Heart rate variability is a non-invasive ECG parameter that assesses sympathetic vagal balance at the sinoatrial level, but its physiological interpretation is still under discussion (104–106). In patients with HF, when short-term or 24-h recordings were analyzed, reduced HRV was consistently observed and explained primarily because of sympathetic and reduced vagal modulation of sinus node (104). For the time domain, the following indexes were calculated according to the recommended values (104): mean n-n interval (SDNN), root mean square difference of successive N–N intervals (RMSSD), Standard deviation of all 5-min mean normal RR intervals (SDANN), Mean RR interval for 24 h (mean NN) and proportion of differences between adjacent N–N intervals of > 50 ms (PNN50). In an 8-week, open-label, prospective clinical trial (NCT01392560) (97), EMPA increased RMSSD and SDNN, but not statistically significant. In the EMBODY trial (107), SDNN (101.1S vs. 112.8S, *P* < 0.01), SDANN (81.0S vs. 92.7S, *P* = 0.02), and RMSSD (34.2S vs. 40.7S, *P* = 0.01) were significantly higher after 24 weeks of EMPA versus baseline.

## Limitations

There are some limitations in this paper. We analyze all the articles on EMPA for HF from the WOSCC database, but due to differences in text quality, the credibility of the analysis may be low. With the continuous updating of WOSCC literature, the number of literatures obtained is different from the actual number of literatures, and the cited times and H-index will also change accordingly.

## Conclusion

The research progress, hotspots, and frontier in this field in the decade were elucidated by us through information visualization technology. The publications of manuscripts were on a rise. With regards to country, USA leads the field in research maturity. The scholars, institutions, and representative literature that play an important role in this area have been identified. The current research focuses on the following three aspects: EMPA improves LV remodeling, exert renal protection, and increases HRV.

## Data availability statement

The original contributions presented in this study are included in the article/supplementary material, further inquiries can be directed to the corresponding author.

## Author contributions

XZ and YZ designed the study and co-wrote the manuscript. XZ re-examined and analyzed the data. YH reviewed and revised the manuscript. All authors contributed to the article, approved the submitted version, and agreed to be held responsible for all aspects of the work.

## Footnotes

1
https://clinicaltrials.gov/ct2/home
